# Application of Multi-Species Microbial Bioassay to Assess the Effects of Engineered Nanoparticles in the Aquatic Environment: Potential of a Luminous Microbial Array for Toxicity Risk Assessment (LumiMARA) on Testing for Surface-Coated Silver Nanoparticles

**DOI:** 10.3390/ijerph120708172

**Published:** 2015-07-15

**Authors:** YounJung Jung, Chang-Beom Park, Youngjun Kim, Sanghun Kim, Stephan Pflugmacher, Seungyun Baik

**Affiliations:** 1 Environmental Safety Group, Korea Institute of Science and Technology Europe (KIST Europe) Forschungsgesellschaft mbH, Universitaet des Saarlandes Campus E7 1, Saarbruecken 66123, Germany; E-Mails: yjjung@kist-europe.de (Y.J.); cb.park@kist-europe.de (C.-B.P.); youngjunkim@kist-europe.de (Y.K.); shkim@kist-europe.de (S.K.); 2 Institute of Ecology, Chair Ecological Impact Research and Ecotoxicology, Technische Universität Berlin, Ernst-Reuter-Platz 1, Berlin 10587, Germany; E-Mail: stephan.pflugmacher@tu-berlin.de

**Keywords:** surface-coated silver nanoparticles (AgNPs), salt stability, surface charge, ecotoxicological screening test, multi-species of luminescent bacteria

## Abstract

Four different manufactured surface-coated silver nanoparticles (AgNPs) with coating of citrate, tannic acid, polyethylene glycol, and branched polyethylenimine were used in this study. The toxicity of surface-coated AgNPs was evaluated by a luminous microbial array for toxicity risk assessment (LumiMARA) using multi-species of luminescent bacteria. The salt stability of four different AgNPs was measured by UV absorbance at 400 nm wavelength, and different surface-charged AgNPs in combination with bacteria were observed using scanning electron microscopy (SEM). Both branched polyethylenimine (BPEI)-AgNPs and polyethylene glycol (PEG)-AgNPs were shown to be stable with 2% NaCl (non-aggregation), whereas both citrate (Cit)-AgNPs and tannic acid (Tan)-AgNPs rapidly aggregated in 2% NaCl solution. The values of the 50% effective concentration (EC_50_) for BPEI-AgNPs in marine bacteria strains (1.57 to 5.19 mg/L) were lower than those for the other surface-coated AgNPs (*i.e.*, Cit-AgNPs, Tan-AgNPs, and PEG-AgNPs). It appears that the toxicity of AgNPs could be activated by the interaction of positively charged AgNPs with the negatively charged bacterial cell wall from the results of LumiMARA. LumiMARA for toxicity screening has advantageous compared to a single-species bioassay and is applicable for environmental samples as displaying ranges of assessment results.

## 1. Introduction

Bioassays using bacteria for toxicity screening have been applied widely as acute toxicity bioassays to understand the ecotoxicological impact of pollutants on aquatic organisms [1,2] due to their advantages in simplicity, rapidity, cost-efficiency, and reproducibility [2,3,4,5]. The microbial assay for toxicity risk assessment (MARA) has recently been developed for the ecotoxicological assessment of chemical and environmental samples [6,7]. This assay was evaluated by comparison with other bioassays, such as invertebrate (*Daphnia magna* and *Thamnocephalus platyurus*) microbiotests, and Microtox^®^, which uses the luminescent bacteria *Vibrio fischeri* as a bacterial strain [8]. MARA, a multi-species assay, uses 11 varying microbial strains that show different sensitivities to different chemicals. This results in the array producing 11 inhibition values indicative of biological toxicity for the tested chemicals or samples [6,7,8]. This assay has some advantageous compared to the other microbiotests for the initial assessment of toxicity for a number of chemicals and environmental samples at various sensitivity ranges [7].

A bioassay that uses luminescent bacteria such as *Vibrio fischeri* could be widely used to test a variety of environmental samples including surface and groundwater samples as well as municipal wastewater effluents and sediments. This assay could be applied as a sensitive and rapid screening tool to evaluate the whole of effluent toxicity [9]. As mentioned, Microtox^®^ is a commercially available toxicological assessment tool that uses a luminescent bacterial strain of *Vibrio fischeri*. It uses a unique software, MicrotoxOmni, to analyze the toxicity [10]. This is one of the most commonly used ecotoxicological assessment tools due to its standardized protocol, globally available materials, and lack of complicated preculturing steps of test biota. However, Microtox^®^ uses only a single bacterial strain, which limits the various sensitivity ranges when compared to the other assessment tools with multi-species of bacteria such as MARA. To overcome this limitation, a luminous microbial array for toxicity risk assessment (LumiMARA) has recently been developed. This bioassay uses 11 different luminescent bacteria cultured from both marine and freshwater. Compared to MARA, LumiMARA provides more sensitive results for the toxicity assessment of chemicals and environmental samples. Moreover, because LumiMARA is a multi-species microbial bioassay, toxicity assessment results have various sensitivity ranges compared to the other toxicity assessment tools such as Microtox^®^ that use a single luminescent bacteria strain.

With rapidly developing nanotechnology, applications and uses of nanomaterials (NMs) are emerging in many fields of industry (e.g., biomedical, electronic, construction, *etc.*) [11]. Although NMs are defined as materials with one dimension less than 100 nm, nanoparticles (NPs) are defined as materials with at least two dimensions between 1 and 100 nm. Among the groups of NMs, nanoparticles (NPs) are particularly important in their applications and usages [12]. Among all anthropogenic NPs, interest in silver NPs (AgNPs) is growing with the usage of AgNPs in consumer becoming more common [13,14,15] for the potential biocidal effects of silver ions and their nanoparticulated form [16,17,18,19,20]. Increasing the use of AgNPs could also lead to more environmental discharge and potentially serious risks [14,21], because the release of AgNPs to the aquatic environment cannot be completely avoided when evaluating their potential antimicrobial properties [15]. Toxicological assessment of these AgNPs in the aquatic environment has been performed by various methods including bacteria, fungi, mammalian cells, and invertebrates [21,22]. These methods, however, may need much effort to understand the toxic mechanisms on tested organisms. Despite the importance of understanding for toxic mechanisms in depth, evaluation including acute and chronic ecotoxicological tests should be performed by easy and reliable screening methods. In addition, both quantitative and qualitative monitoring of environmental pollutants including NPs is also important. Quantitative analysis of NPs including AgNPs in the aquatic environment is in a big challenge, and thus qualitative assessment in regarding toxicological effects with bioassay could be alternative.

In this study, therefore, several surface-modified AgNPs were selected as target compounds for ecotoxicological assessment with multi-species luminescent bacteria bioassay. Surface coating technology of NPs can usually be applied to AgNPs to increase their stabilization in suspension [23,24], and four different surface-coated AgNPs were selected for ecotoxicological assessment by LumiMARA. Elucidation of the practicability for LumiMARA as multi-species bioassay will be presented as the results of this study and application of this bioassay for environmental samples will then be discussed.

## 2. Materials and Methods

### 2.1. Surface-Coated Silver Nanoparticles (AgNPs)

Four spherical AgNPs with an average particle size of 20 nm were obtained from Nanocomposix (San Diego, CA, USA). The AgNPs had surface coating materials of citrate (cit), tannic acid (tan), polyethylene glycol (PEG), and branched polyethylenimine (BPEI). These materials were selected based on different physicochemical properties of salt stability and zeta potential. Their physicochemical properties are listed in Table 1.

### 2.2. Luminous Microbial Array for Toxicity Risk Assessment (LumiMARA)

The values of the 50% effective concentration (EC_50_) for bioluminescent bacteria exposed to four different AgNPs were measured following the manufacturer’s protocol using Luminous microbial assay for toxicity risk assessment (LumiMARA) system (NCIMB Ltd., Bucksburn, Aberdeen, UK), including 11 freeze-dried bioluminescent bacteria consisting of 9 marine bacteria and 2 freshwater bacteria (Table 2). Briefly, 20 mg/L of AgNPs, the highest concentration tested, were prepared and filtered through a 0.2-µm sterile syringe filter (Whatmann, Buckinghamshire, UK) into a clean sterile vessel. Additionally, 2% NaCl were added and dissolved to the 20 mg/L of AgNPs to be tested with 9 marine bacteria, but not with 2 fresh bacteria. Freeze-dried bioluminescent bacteria were allowed to stand at room temperature for l h to equilibrate before reconstituting with the provided reagents, 100 µL of osmotic adjusting solution for 9 marine bacteria (Reagent 1) and 100 µL of diluent for 2 freshwater bacteria (Reagent 2), followed by pre-incubating for 15 min at 28 °C. To assess the toxicity of AgNPs and their concentration-dependent inhibition of bacteria growth, 11 individual bacteria strains were exposed to target AgNPs at concentrations of 0, 1.25, 2.5, 5, 10, and 20 mg/L for 15 minutes at 28 °C. After 15 min of incubation, they were read by a luminometer (Tristar^2^ LB 942 Multimode Reader, Berthold Technologies, Bad Wildbad, Germany). The toxicological effects of AgNPs on bioluminescent bacteria were then quantified by the reduction of light from the luminescent bacteria.

**Table 1:** Physicochemical properties of silver nanoparticles (AgNPs) coated with different capping agents of citrate (Cit), tannic acid (Tan), polyethylene glycol (PEG), and branched polyethylenimine (BPEI).

	Cit-AgNP	Tan-AgNP	PEG-AgNP	BPEI-AgNP
Shape	Sphere	Sphere	Sphere	Sphere
Particle surface	Sodium citrate	Tannic acid	PEG	BPEI
D_h_ (nm) *	23.9 ± 0.07	25.9 ± 0.13	44.3 ± 4.30	64.6 ± 5.29
PdI *	0.11 ± 0.01	0.20 ± 0.02	0.26 ± 0.08	0.25 ± 0.02
ζ potential (mV) *	−34.9 ± 2.95	−41.4 ± 2.70	−16.8 ± 3.51	16.7 ± 3.13
pH	7.00 ± 0.10	6.91 ± 0.01	6.99 ± 0.01	7.04 ± 0.00
SEM image **	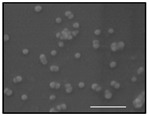	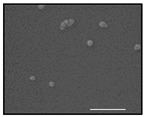	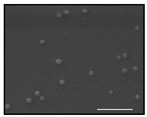	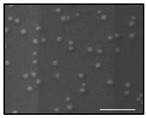
Structure of capping agent	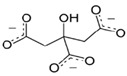	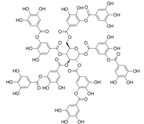	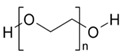	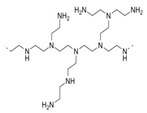

* Hydrodynamic diameter, polydispersity index (PdI), and zeta potential were analyzed by Malvern Zetasizer Nano ZS at 25 °C with the 173° of a back-scattering angle using 10 mg/L of AgNPs in MilliQ water, respectively. ** Dispersion and morphology of each AgNPs were observed by Scanning Electron Microscopy (SEM) images, using FEI Quanta 250 FEG scanning electron microscope (Scale bar = 200 nm).

**Table 2:** 11 Bioluminescent bacteria strains for luminous microbial assay for toxicity risk assessment (LumiMARA).

LumiMARA Number	Luminescent Bacteria Strains		
#1	*Photobacterium leiognathi*	(NCIMB 30266)	Marine bacteria (Order: Vibrionales)
#2	*Photobacterium phosphoreum*	(NCIMB 30267)	
#3	*Vibrio fischeri*	(NCIMB 30268)	
#4	*Photobacterium leiognathi*	(NCIMB 30269)	
#5	*Photobacterium phosphoreum*	(NCIMB 30270)	
#6	*Photobacterium phosphoreum*	(NCIMB 30271)	
#7	*Vibrio harveyi*	(NCIMB 30272)	
#8	*Vibrio harveyi*	(NCIMB 30273)	
#9	*Vibrio fischeri*	(NCIMB 30274)	
#10	*Photorhabdus luminescens*	(NCIMB 30275)	Freshwater bacteria (Order: Enterobacteriales)
#11	*Photorhabdus asymbiotica*	(NCIMB 30276)	

### 2.3. UV-Vis Spectrometer

Four different AgNPs were prepared in both pure MilliQ water (EMD Millipore, Billerica, MA, USA) and Milli-Q water containing 2% NaCl to adjust samples to conditions for marine bacteria growth. Aggregation of surface coated AgNPs, which is related to their salt stability, was observed by a double beam UV-Vis spectrometer with deuterium and tungsten-halogen lamps (Lambda 35, Perkin-Elmer, Waltham, MA, USA) and recorded as the absorption spectra between 190 and 1100 nm.

### 2.4. Scanning Electron Microscopy (SEM)

The aggregation of AgNPs and the damage to luminescent bacteria strains after exposure to the AgNPs were observed by SEM using a Quanta 250 FEG (FEI Company, Eindhoven, Holland) at an accelerating voltage of 30.0 and 8.00 kV, respectively. To investigate the fate of the AgNPs in the tested media, each 50 mg/L AgNPs sample in both the pure Milli-Q water and the 2% NaCl solution was centrifuged at 8000 rpm for 10 min. In addition, to test the AgNPs exposure damage, luminescent bacteria strains exposed to each prepared AgNPs at a concentration of 10 mg/L for 15 min at 28 °C. After 15 min of incubation, samples were centrifuged at 10,000 rpm for 15 min. After supernatant was decanted, the settled bacterial pellets by precipitation were fixed with 2% glutaraldehyde (Sigma-Aldrich, St. Louis, MO, USA) for 2 h at room temperature before being dehydrated in serial concentrations of ethanol from 10% to 100%. Then, 15 µL of each sample was put on a silicon wafer and air-dried overnight at room temperature. All AgNPs and bacteria samples were sputter coated using platinum at 1 × 10^−3^ and 6 × 10^−^^3^ mbar for 40 and 15 s, respectively, with a deposition current of 120 mA using a K675X sputter coater (EMITECH, Ashford Kent, UK) to prevent the accumulation of an electrostatic charge on the surface.

### 2.5. Statistical Analysis

The values of EC_50_ for bioluminescent bacteria exposed to four different AgNPs were calculated from fitted sigmoidal dose-response curves using OriginPro software (OriginPro version 8.0, Northampton, MA, USA) and the all dose-response curves are shown in Figures S1 to S11 (Supplementary Information). All tests were performed in sextuplicate with experiments conducted in triplicate on two different days. All data are shown as the average of these six samples with 95% confidence intervals. Comparison of the effective concentration data was carried out using a *post hoc* Fisher’s test in the one-way ANOVA (OriginPro version 8.0, Northampton, MA, USA). Statistical significance was set at *p* < 0.05.

## 3. Results and Discussion

### 3.1. Aggregation of Surface-Coated Silver Nanoparticles (AgNPs) in 2% NaCl Solution

**Figure 1:**
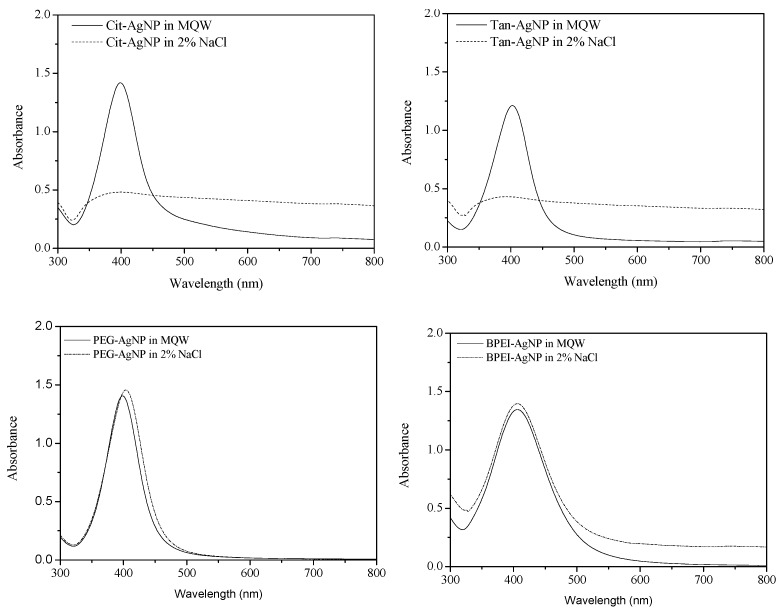
UV-Vis absorption spectrum peaks of surface-coated AgNPs in both MilliQ water only (straight line) and 2% NaCl solution (dashed line).

**Figure 2:**
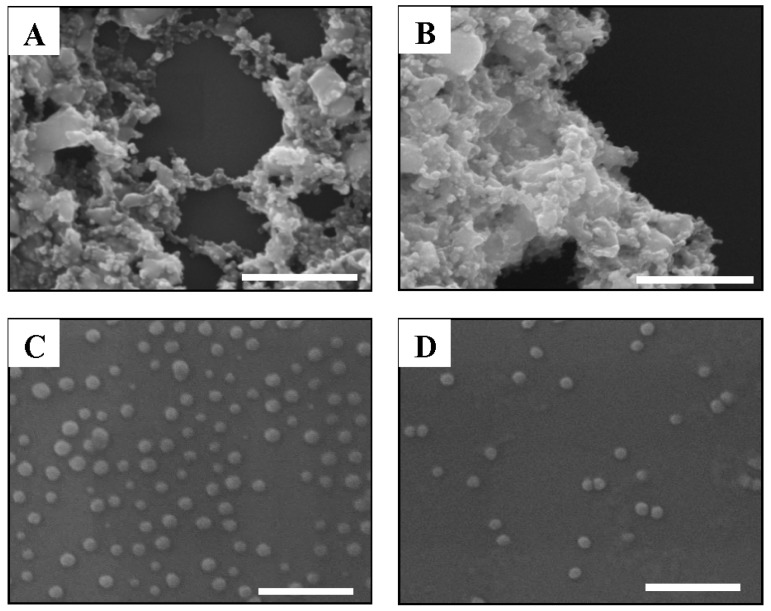
SEM images of surface-coated AgNPs in 2% NaCl solution. (**A** and **B**) Aggregation of both citrate (Cit)-AgNPs and tannic acid (Tan)-AgNPs in 2% NaCl solution and (**C** and **D**) non-aggregation of both polyethylene glycol (PEG)-AgNPs and branched polyethylenimine (BPEI)-AgNPs in 2% NaCl solution. (Scale bar = 200 nm)

UV-Vis spectroscopy was used not only to analyze the surface plasmon resonance of nanoparticles with the absorption spectrum peak between 400 and 450 nm [25,26] but also to quantify the aggregation rates of NPs in solution [27]. In this study, the aggregation of AgNPs in 2% NaCl solution was measured with the UV-Vis spectrum to examine the salt stability of surface-coated AgNPs (Figure 1 and Figure 2) since 2% NaCl solution was used for culturing media of marine luminescent bacteria. UV absorption spectrum peaks of surface-coated AgNPs in 2% NaCl solution are shown in Figure 1. As shown in this figure, UV-Vis absorption peaks of both PEG-AgNPs and BPEI-AgNPs in 2% NaCl solution were observed between 399 and 405 nm, which are similar to those of surface-coated AgNPs in pure MilliQ water. However, the UV absorption peaks of Cit-AgNPs and Tan-AgNPs were not observed in 2% NaCl solution. The aggregation of Cit-AgNPs and Tan-AgNPs in 2% NaCl solution was also clearly verified by SEM images (Figure 2A,B), whereas the morphological shapes of PEG-AgNPs and BPEI-AgNPs were only slightly changed in the same solution (Figure 2C,D). The surfaces of Cit-AgNPs and Tan-AgNPs were negatively charged by coating materials of citrate and tannic acid, which enhance stability of nanoparticles suspension by electrostatic repulsion [28]. The aggregation phenomenon of both Cit-AgNPs and Tan-AgNPs in 2% NaCl solution can be explained by DLVO (*Derjaguin*-*Landau*-*Verwey*-*Overbeek*) theory, which describes the stability of particles in a liquid medium as the sum of the van der Waals attractive force and double layer repulsive force [29,30]. Therefore, it seems that aggregations of Cit-AgNPs and Tan-AgNPs in 2% NaCl solution were caused by the disruption of the electrostatic repulsion. Contrarily, polymer based coating materials, PEG and BPEI in this study, may have a strong adsorption on the surface of AgNPs to have the steric repulsion as well as the electrostatic repulsion, and these repulsion forces may lead better stabilization of PEG-AgNPs and BPEI-AgNPs in 2% NaCl solution compared to Cit-AgNPs and Tan-AgNPs [31]. El Badawy *et al.* investigated the aggregation kinetics of AgNPs with different coating materials and resulted in the critical aggregation of Cit-AgNPs with 70 mM NaCl solution but in stabilization of BPEI-AgNPs with up to 1000 mM NaCl solution [32]. Based on UV-Vis absorption peaks and SEM images obtained from this study, we suggest that both Cit-AgNPs and Tan-AgNPs were rapidly aggregated in 2% NaCl solution, but both PEG-AgNPs and BPEI-AgNPs were very stable in the same solution.

### 3.2. Toxicity Screening Test Using a Luminous Array for Toxicity Risk Assessment (LumiMARA)

Toxicity screening tools for various pollutants should be sensitive, rapid, and cost effective [9]. Luminescent bacteria are frequently used as a test organism for toxicity screening of environmental pollutants [1,3,5,33]. Among the currently available toxicity screening tools, Microtox^®^ is well known as a suitable tool for toxicity screening of pollutants with a single luminescent bacteria, *Vibrio fischeri* [10,34]. In this study, the LumiMARA system using multi-species of luminescent bacteria was introduced as a toxicity screening tool of AgNPs to better understand the environmental toxicological effects of AgNPs [7].

#### 3.2.1. The Values of Effective Concentrations of Surface-Coated Silver Nanoparticles (AgNPs)

**Figure 3:**
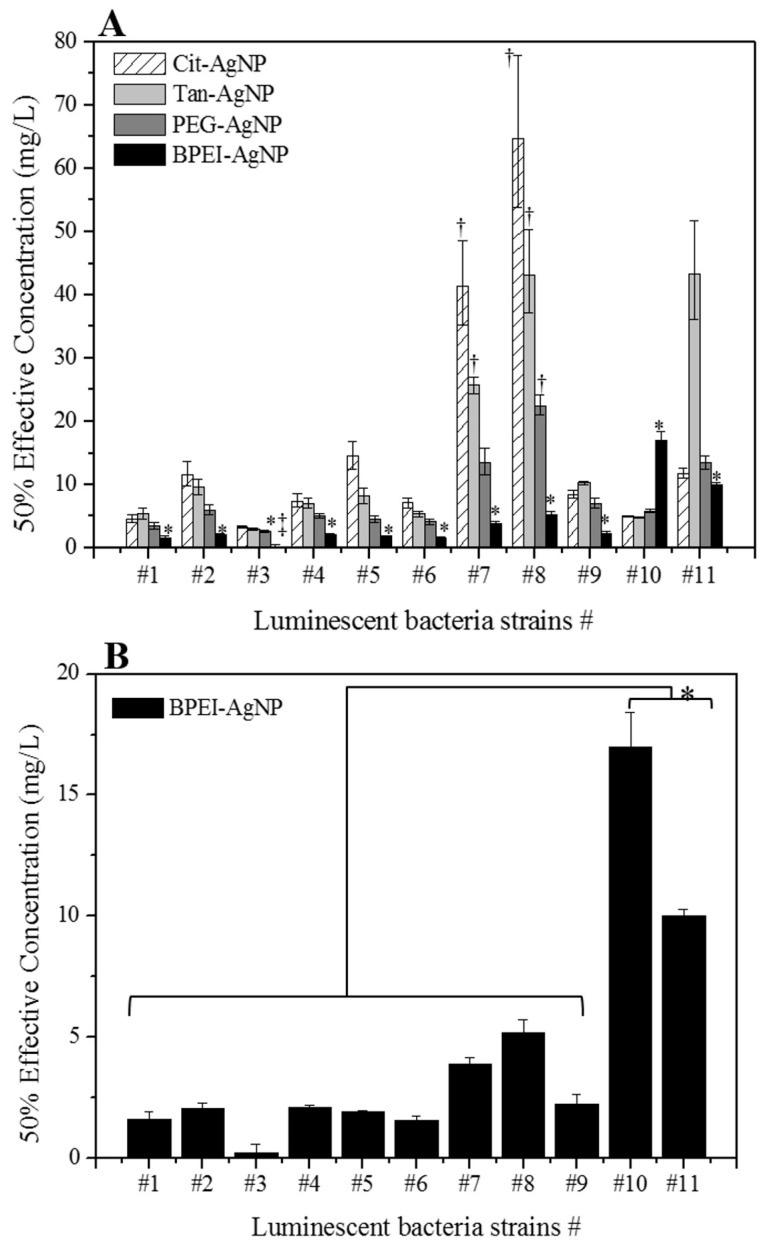
The 50% effective concentrations (EC_50_) in 11 luminescent bacteria strains exposed to surface-coated AgNPs. (**A**) EC_50_ values for four different AgNPs and (**B**) EC_50_ values for BPEI-AgNPs. All EC_50_ values represent average with 95% confidence intervals (*n* = 6). ‡ denotes EC_50_ is lower than the lowest dose concentration in this study (1.25 mg/L). † denotes EC_50_ is higher than the highest dose concentration in this study (20.0 mg/L). * denotes significant differences between all luminescent bacteria strains exposed to AgNPs.

The 50% effective concentrations (EC_50_) of four different surface-coated AgNPs exposed to 11 luminescent bacteria are shown in Figure 3 and Table 3. Among all tested AgNPs, the EC_50_ values for BPEI-AgNPs were significantly lower for marine luminescent bacteria, strains #1 to #9 at 1.57 mg/L (1.42–1.73 mg/L with 95% of CI) to 5.19 mg/L (4.71–5.73 mg/L with 95% CI), while the other surface-coated AgNPs showed 4.57 mg/L (4.02–5.19 mg/L with 95% of CI) to 64.8 mg/L (53.9–77.8 mg/L with 95% of CI) for Cit-AgNPs, 2.94 mg/L (2.80–3.08 mg/L with 95% of CI) to 43.2 mg/L (37.1–50.4 mg/L with 95% of CI) for Tan-AgNPs, and 2.59 mg/L (2.42–2.77 mg/L with 95% of CI) to 22.5 mg/L (21.0–24.1 mg/L with 95% of CI) for PEG-AgNPs (Figure 3A and Table 3). In particular, the EC_50_ value of BPEI-AgNPs for marine bacterial strain #3, *Vibrio fischeri* (NCIMB 30268), was calculated as 0.216 mg/L from its dose-response curve, suggesting that the EC_50_ value of BPEI-AgNPs was lower than 1.25 mg/L, the lowest dose concentration in this study. This result at the lowest concentration indicates that bacterial strain #3 is the most sensitive organism to BPEI-AgNPs. On the other hand, the EC_50_ values for Cit-AgNPs on freshwater bacteria strains #10 and #11 (4.99 to 11.8 mg/L, respectively, *p* < 0.05, Figure 3B and Table 3) were significantly lower than that of BPEI-AgNPs or similar (10.0 to 17.0 mg/L, respectively, *p* < 0.05, Figure 3B and Table 3). These results indicate that the toxicity of BPEI-AgNPs is higher in the marine environment than in a freshwater environment because the EC_50_ value of BPEI-AgNPs was significantly higher in freshwater bacteria than marine bacteria. In contrast, it is possible that the toxicity of Cit-AgNPs may be increased in a freshwater environment compared to the marine environment. In addition, the EC_50_ values for Tan-AgNPs, including some of the EC_50_ values for Cit-AgNPs and/or PEG-AgNPs, could not be calculated experimentally for the #7, #8, and #11 bacteria strains because these EC_50_ values were higher than the highest dosing concentration (20 mg/L) in this study (Figure 3A and Table 3). These results suggest that #7 and #8 marine bacteria strains and the #11 freshwater bacteria strain were not sensitive to Tan-AgNPs including Cit-AgNPs and/or PEG-AgNPs.

**Table 3:** The values of 50% effective concentration (EC_50_) in 11 bioluminescent bacteria exposed to four different surface-coated AgNPs.

Average Values of EC_50_ (95% CI) (mg/L, N = 6)					
Luminescent Bacteria Strains	Cit-AgNPs	Tan-AgNPs	PEG-AgNPs	BPEI-AgNPs	Species
#1	4.57 (4.02 – 5.19)	5.40 (4.58 – 6.37)	3.44 (2.93 – 4.05)	1.62 (1.37 – 1.91)	Marine bacteria
#2	11.6 (9.80 – 13.7)	9.60 (8.44 – 10.9)	5.95 (5.24 – 6.76)	2.07 (1.87 – 2.29)	
#3	3.36 (3.18 – 3.55)	2.94 (2.80 – 3.08)	2.59 (2.42 – 2.77)	0.216 ^‡^ (0.0832 – 0.560)	
#4	7.41 (6.44 – 8.53)	7.07 (6.37 – 7.85)	5.07 (4.68 – 5.49)	2.08 (1.97 – 2.20)	
#5	14.5 (12.4 – 16.9)	8.15 (7.02 – 9.47)	4.50 (3.91 – 5.17)	1.92 (1.88 – 1.96)	
#6	7.10 (6.36 – 7.91)	5.37 (4.94 – 5.85)	4.10 (3.74 – 4.50)	1.57 (1.42 – 1.73)	
#7	41.4 ^†^ (35.3 – 48.5)	25.7 ^†^ (24.4 – 27.0)	13.5 (11.5 – 15.8)	3.87 (3.61 – 4.15)	
#8	64.8 ^†^ (53.9 – 77.8)	43.2 ^†^ (37.1 – 50.4)	22.5 ^†^ (21.0 – 24.1)	5.19 (4.71 – 5.73)	
#9	8.49 (7.97 – 9.05)	10.3 (10.0 – 10.6)	7.02 (6.26 – 7.86)	2.24 (1.91 – 2.64)	
#10	4.99 (4.81 – 5.17)	4.80 (4.71 – 4.88)	5.83 (5.54 – 6.12)	17.0 (15.7 – 18.4)	Freshwater bacteria
#11	11.8 (11.0 – 12.7)	43.3 ^†^ (36.2 – 51.8)	13.5 (12.4 – 14.6)	10.0 (9.81 – 10.3)	

^‡^ EC_50_ is lower than 1.25 mg/L, which was the lowest dose concentration in this study. ^†^ EC_50_ is higher than 20 mg/L, which was the highest dose concentration in this study.

#### 3.2.2. Methodological Approach to Evaluate the Toxicity of AgNPs

**Figure 4:**
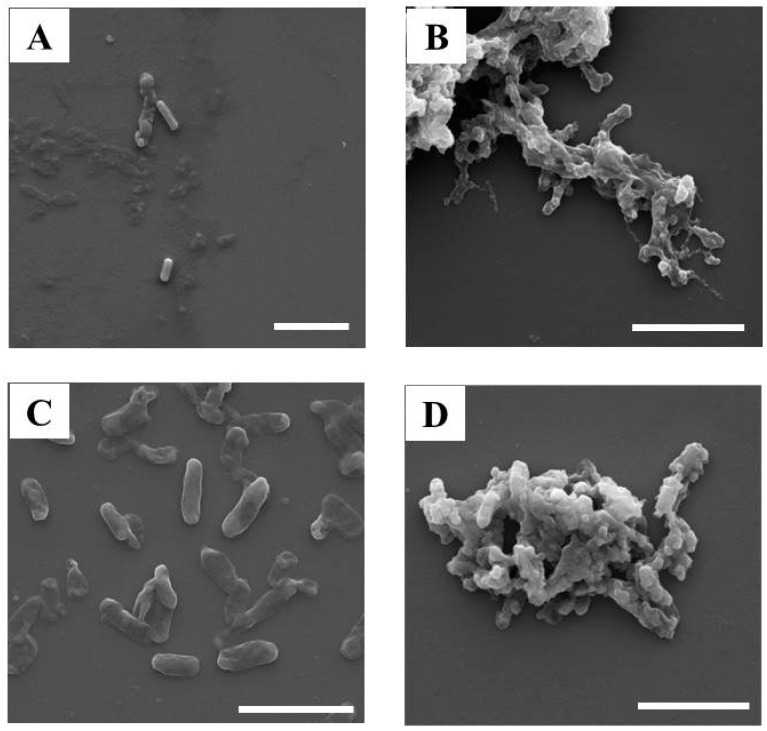
SEM images of Cit-AgNPs and BPEI-AgNPs in marine bacteria strain #3 (<1.25 mg/L of EC_50_ value for BPEI-AgNPs) and strain #8 (>20 mg/L of EC_50_ value for Cit-AgNPs). (**A**) Marine bacteria strain #3 exposed to Cit-AgNPs (not adhesion); (**B**) Marine bacteria strain #3 exposed to BPEI-AgNPs (adhesion with bacteria); (**C**) Marine bacteria strain #8 exposed to Cit-AgNPs (not adhesion); (**D**) Marine bacteria strain #8 exposed to BPEI-AgNPs (adhesion with bacteria). (Scale bar = 5 µm)

The results of ecotoxicological assessment were obtained from the EC_50_ values with LumiMARA (Figure 3 and Table 3). Unfortunately, modes of toxic action could not be determined by the results obtained from the toxicity test with LumiMARA itself. In addition, the concentrations of free silver ions released from all tested AgNPs were not significant as the results of the titration method with the ion selective electrode (Supplementary Information), because free silver ions released from all tested AgNPs were not detectable with the detection limit of 0.01 mg/L, which is lower than the minimum lethal concentration of silver ions, 0.025 mg/L, on different gram negative bacteria, *Escherichia coli*, from the other gram negative bacteria, luminescent bacteria, applied in this study [35]. This indicates that the effects of free silver ions released from all tested AgNPs on luminescent bacteria, gram-negative bacteria, were limited in this study. Therefore, the correlation between physicochemical properties and toxicity of the AgNPs was evaluated by SEM image observation (Figure 4) in this study, with particular attention paid to BPEI-AgNPs and Cit-AgNPs. For this observation, strains of #3 *Vibrio fischeri* (EC_50_ values for BPEI-AgNPs and Cit-AgNPs are <1.25 mg/L and 3.36 mg/L, respectively) and #8 *Vibrio harveyi* (EC_50_ values for BPEI-AgNPs and Cit-AgNPs are 5.19 mg/L and >20.0 mg/L, respectively), which are the most and the least sensitive strain among the tested bacteria for both BPEI-AgNPs and Cit-AgNPs, were selected. As shown in Figure 4, the bacteria exposed to Cit-AgNPs exhibited a rod shape with an average length of 2 µm (Figure 4A,C), while the bacteria exposed to BPEI-AgNPs were aggregated (Figure 4B,D). Bacterial growth inhibition of up to 98% at 2.0 µg/mL concentration was also induced by the positively charged BPEI-AgNPs mainly due to the interaction between cationic polymers and the negatively charged cell membrane [36]. Morones *et al.* suggested three toxic mechanisms by AgNPs on the bacteria: (1) attachment of AgNPs on the surface of bacterial cell wall which contains sulfur-containing protein to have a tendency to react with AgNPs; (2) penetration of AgNPs into the bacteria through the cell membrane to react with sulfur-containing proteins inside of the cell as well as phosphorus-containing DNA; and (3) contribution of silver ions released from AgNPs [37]. With the results from BPEI-AgNPs, the observation of the attachment for BPEI-AgNPs on to the surface of the bacterial cell wall was achieved (Figure 4B,D) but the penetration of AgNPs into the bacteria was unable to perform in this study.

In marine bacteria strains, the toxicity of BPEI-AgNPs was strongly related to the salt stability of nanoparticles in the 2% NaCl solution. Several studies reported that the aggregation of nanoparticles in an aqueous solution is an important factor to elucidate the nanotoxicity in an aquatic environment [38,39,40]. Adverse effects of nanoparticles according to positive or negative surface charges were observed in toxicity tests using various bacteria cells, even if the toxicity mechanism was not examined [23,41,42]. In particular, the aggregation of silver nanoparticles in aqueous solution indicates a loss of nano-characteristics, which influences the toxicity [43]. Stability of nanoparticles in tested medium can also be an important factor. The experimental results on the stability test of surface-coated AgNPs during 15 min of exposure time in tested medium indicate that the effects of changes in the properties of nanoparticles including hydrodynamic diameter with polydispersity index (PdI) and zeta potential on tested bacteria may be limited (Supplementary Information, Figures S3 and S15). Consequently, we suggest that the toxicity of BPEI-AgNPs in marine bacteria strains may be influenced by positively charged surfaces and its stability in 2% NaCl solution. Unfortunately, this suggestion could not be applied to the other surface-coated AgNPs (*i.e.*, Cit-AgNPs, Tan-AgNPs, and PEG-AgNPs). In freshwater bacteria strains, however, the differences of toxicity patterns from the results of marine bacteria strains. Toxicological assessment of four different coating materials was solely performed by the same method with four different surface-coated AgNPs (Figure S14). From the results, different toxicity patterns of coating materials from those of surface-coated AgNPs were found as the most and the least sensitive materials of citrate and BPEI, respectively. This indicates that coating materials may have the effects on the toxicity of surface-coated AgNPs on luminescent bacteria in freshwater. There are some limitations to investigate the toxicological mechanisms of nanoparticles on luminescent bacteria from LumiMARA, but this has potential applicability for the toxicological assessment as a pre-screening tool of nanoparticles in the aquatic environment.

#### 3.2.3. Feasibility of LumiMARA on the Application for Environmental Samples

**Figure 5:**
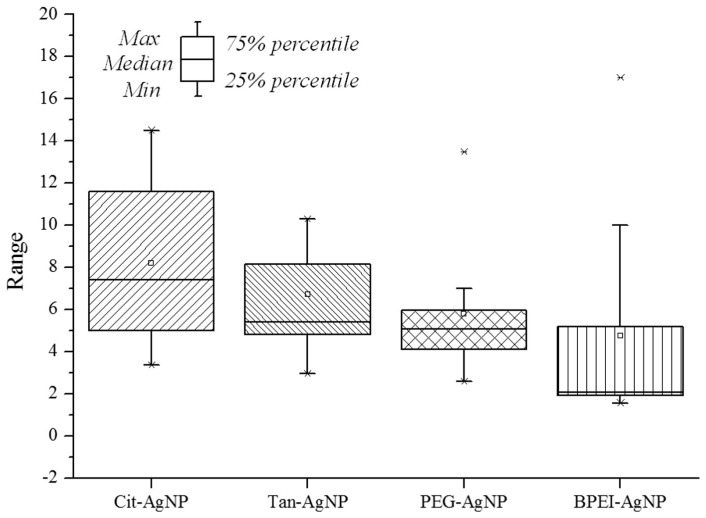
Box plot of average EC_50_ values for four surface-coated AgNPs obtained from LumiMARA experiment; EC_50_ values not achieved experimentally were excluded from this figure.

The exposure time of LumiMARA in this study was 15 min, which has been reported as the optimum exposure time of luminescent bacteria for the toxicological assessment of metals [44,45,46]. This short exposure time of LumiMARA would be drawback for the applicability as the toxicological assessment of nanoparticles, which may have slow toxicity reaction [47]. However, the advantages in simplicity, rapidity, cost-efficiency, and reproducibility of this bioassay may lead to have a potential applicability as an acute toxicity pre-screening tool for the environmental sample containing nanoparticles. Moreover, using multi-species of bacteria for toxicity screening tests can lead to various sensitivity ranges for a number of chemicals and environmental samples. Single bacterial strain toxicity screening tests do not have various sensitivity ranges [7], and thus over-estimation of toxicological results could be achieved. For example, single strain #9 *Vibrio fischeri* (NCIMB 30274) among all other marine luminescent bacteria from LumiMARA showed more sensitive for Cit-AgNPs compared to Tan-AgNPs in this study (Figure 3A). However, the other eight strains showed more sensitive for Tan-AgNPs compared to Cit-AgNPs. Therefore, using bioassay with multi-species of bacteria would be advantageous against using bioassay with single-species of bacteria to have ecotoxicological assessment for environmental samples, which contain complex matrices and various chemicals. Box plots of EC_50_ values of tested luminescent bacteria for four surface-coated AgNPs are depicted in Figure 5. This figure shows the results from the toxicological assessment for each tested nanoparticle explained as a range of outcomes. Specifically, 25% to 75% of the EC_50_ values are shown within the box, and toxicity trends can be compared for the tested nanoparticles. Toxicological assessments with the results displayed as ranges can then provide the feasibility for the testing of environmental samples. Consequently, using this ecotoxicological screening tool with multi-species of luminescent bacteria for real environmental samples will give more realistic toxicological assessment results.

## 4. Conclusions

The stability in aqueous solution and surface charge of AgNPs are important in determining the growth inhibition of multi-species of luminescent bacteria. To identify the safety of nanoparticles, further studies examining the biological responses and ecological effects of the nanoparticles are necessary. Moreover, the fate of AgNPs in the aquatic environment via various non-point and point sources such as wastewater treatment systems is also very important regarding the safety of nanoparticles in the environment. However, using bioassay with multi-species of luminescent bacteria provides the promising results on ecotoxicological screening for AgNPs in the aquatic environment. The combination approach using instrument methods for chemicals analysis and bioassay for ecotoxicological testing would be very important for risk assessment of the aquatic environmental samples, and the bioassay introduced in this study can be applied for this risk assessment in future.

## Supplementary Files

Supplementary File 1
