# Supplementary material for: Application of Multi-Species Microbial Bioassay to Assess the Effects of Engineered Nanoparticles in the Aquatic Environment: Potential of a Luminous Microbial Array for Toxicity Risk Assessment (LumiMARA) on Testing for Surface-Coated Silver Nanoparticles
Source: Int J Environ Res Public Health. 2015 Jul 15;12(7):8172–86. doi: 10.3390/ijerph120708172 (PMC4515715; doi:10.3390/ijerph120708172)
Supplement: Supplementary File 1 [file ijerph-12-08172-s001.pdf]

Supplementary Information

Application of Multi-Species Microbial Bioassay to Assess the Effects of Engineered Nanoparticles in the Aquatic Environment: Potential of a Luminous Microbial Array for Toxicity Risk Assessment (LumiMARA) on Testing for Surface-Coated Silver Nanoparticles

S1. Dose-Response Curves Achieved from the LumiMARA Experiment

The inhibition of each luminescent bacteria strain exposed to the four different surface-coated AgNPs, Cit-AgNPs, Tan-AgNPs, PEG-AgNPs, and BPEI-AgNPs, was quantified by the reduction of light from the luminescent bacteria. The quantified inhibition was then used to develop dose-response curves and fitted with the sigmoidal non-linear function provided by OriginPro version 8.0 (Northampton, MA, USA). The resulting dose-response curves are shown in Figure S1 to S11 for each luminescent bacteria strain used. All tests were performed in sextuplicate with experiments conducted in triplicate on two different days.

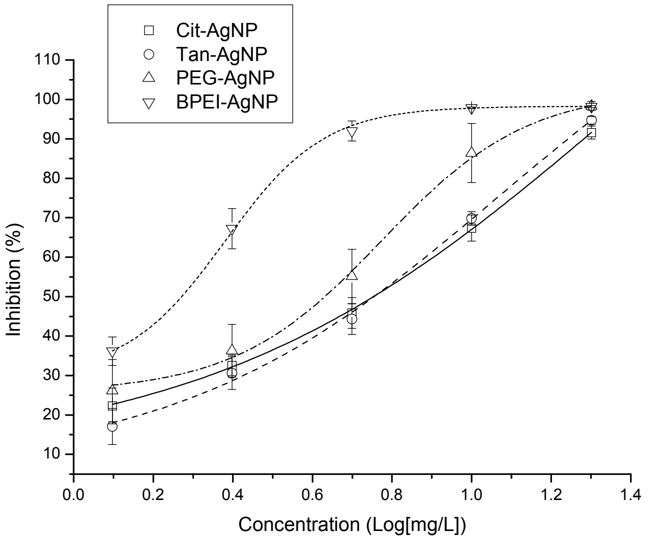

Figure S1. Fitted sigmoidal dose-response curves for the 4 different surface-coated AgNPs with luminescent bacteria strain #1 (*Photobacterium leiognathi*, NCIMB 30266) of the LumiMARA system.

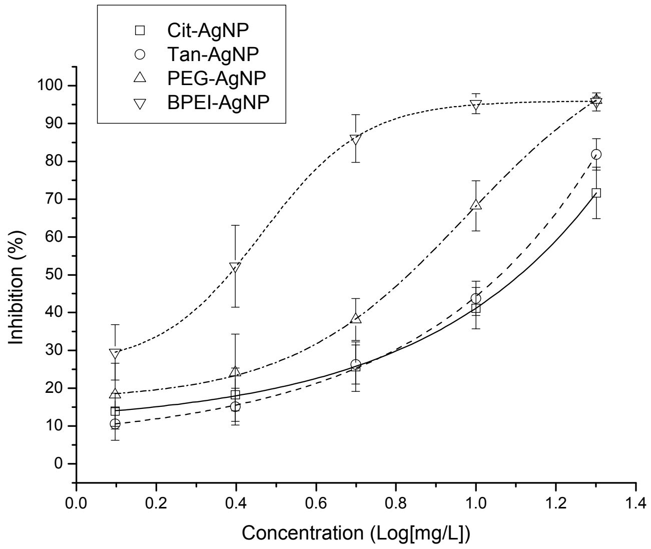

Figure S2. Fitted sigmoidal dose-response curves for the 4 different surface-coated AgNPs with luminescent bacteria strain #2 (*Photobacterium phosphoreum*, NCIMB 30267) of the LumiMARA system.

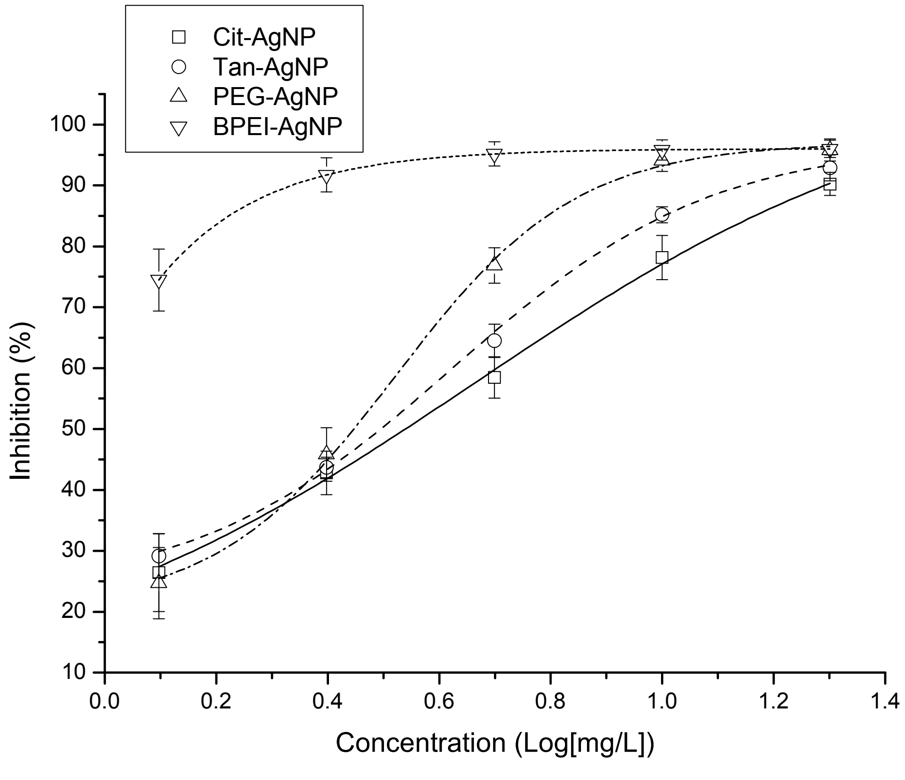

Figure S3. Fitted sigmoidal dose-response curves for the 4 different surface-coated AgNPs with luminescent bacteria strain #3 (*Vibrio fischeri*, NCIMB 30268) of the LumiMARA system.

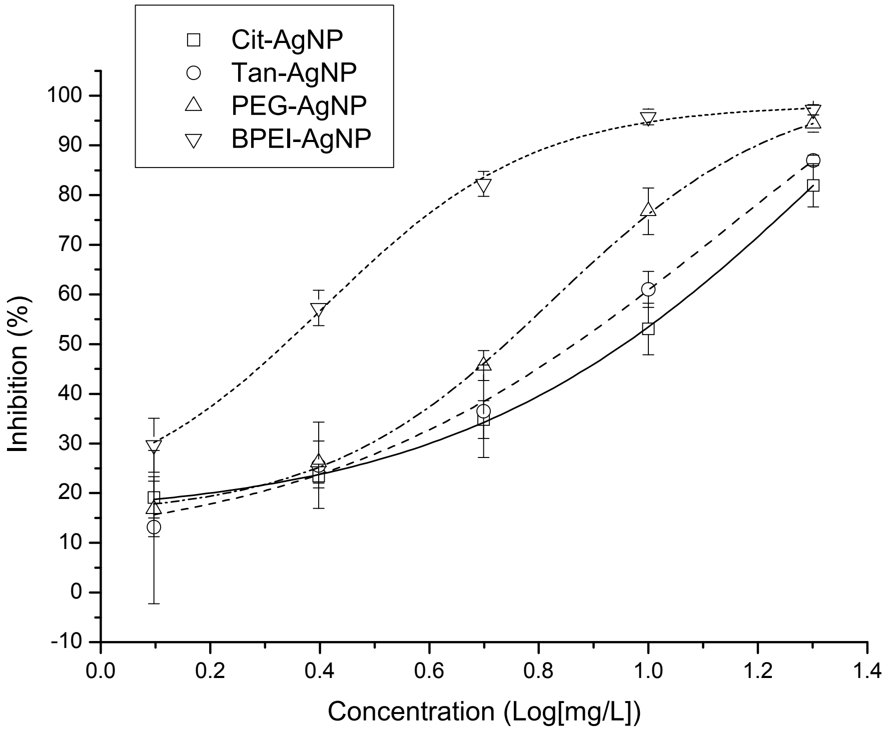

Figure S4. Fitted sigmoidal dose-response curves for the 4 different surface-coated AgNPs with luminescent bacteria strain #4 (*Photobacterium leiognathi*, NCIMB 30269) of the LumiMARA system.

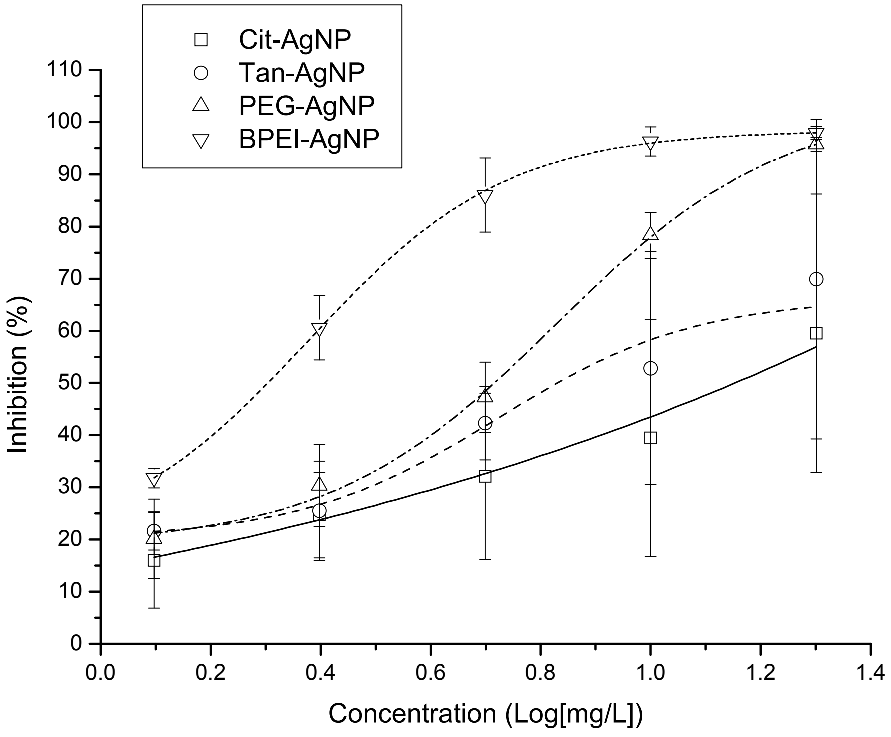

Figure S5. Fitted sigmoidal dose-response curves for the 4 different surface-coated AgNPs with luminescent bacteria strain #5 (*Photobacterium phosphoreum*, NCIMB 30270) of the LumiMARA system.

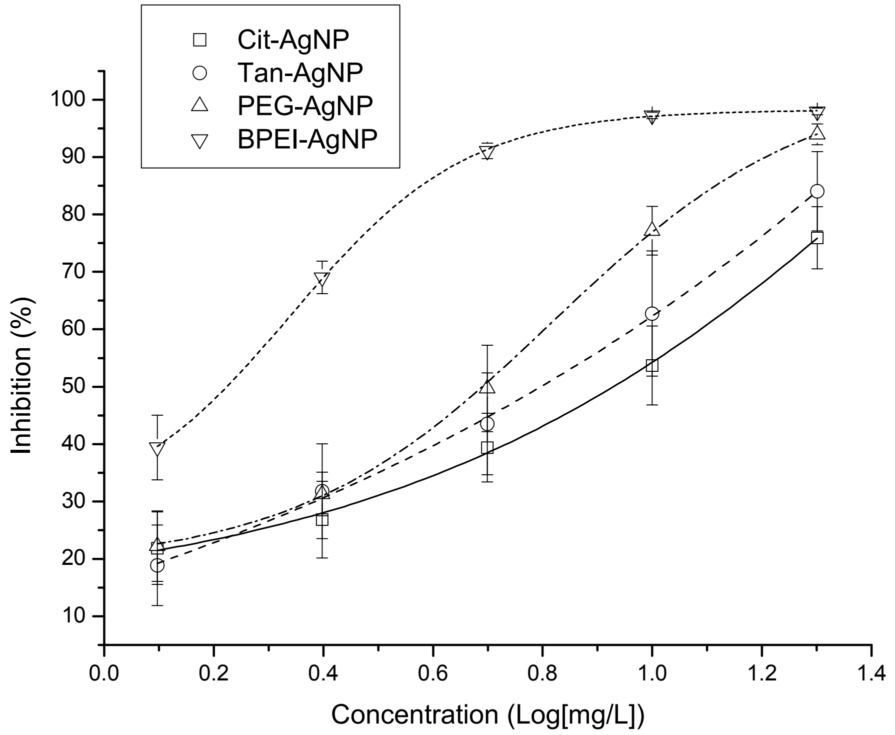

Figure S6. Fitted sigmoidal dose-response curves for the 4 different surface-coated AgNPs with luminescent bacteria strain #6 (*Photobacterium phosphoreum*, NCIMB 30271) of the LumiMARA system.

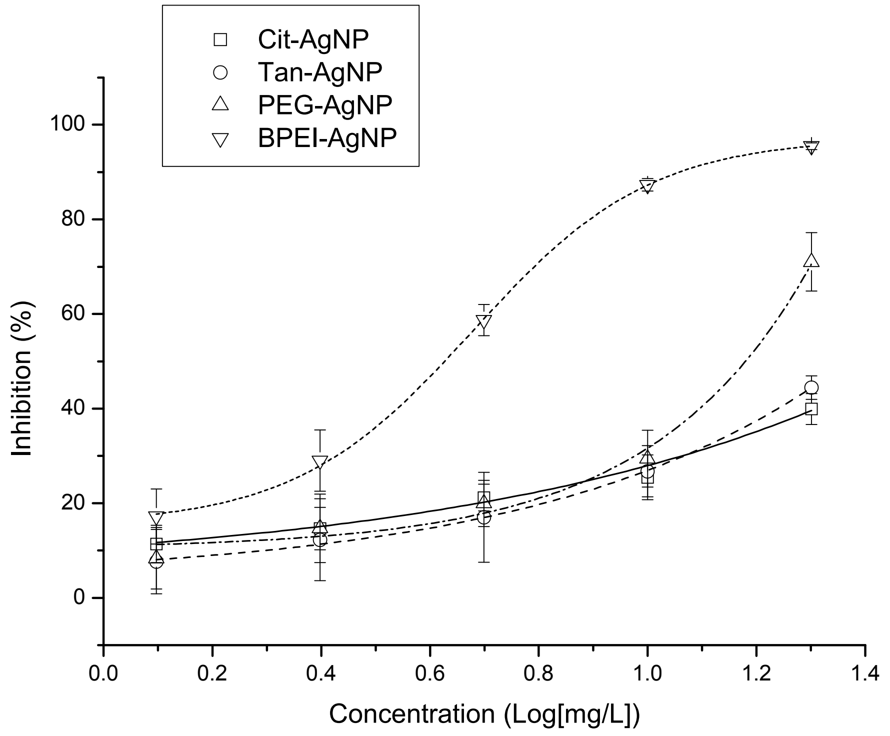

Figure S7. Fitted sigmoidal dose-response curves for the 4 different surface-coated AgNPs with luminescent bacteria strain #7 (*Vibrio harveyi*, NCIMB 30272) of the LumiMARA system.

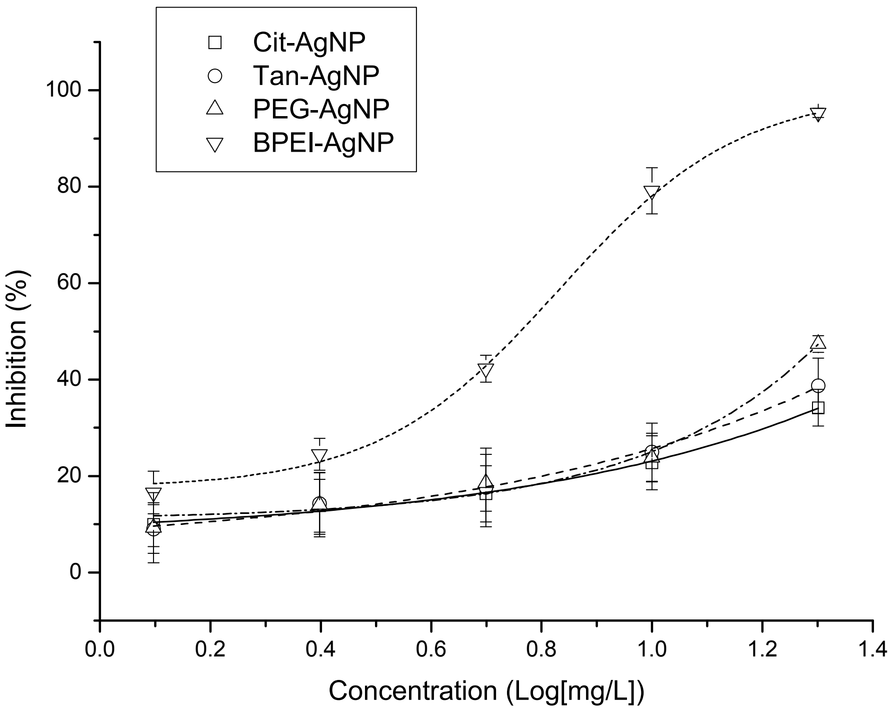

Figure S8. Fitted sigmoidal dose-response curves for the 4 different surface-coated AgNPs with luminescent bacteria strain #8 (*Vibrio harveyi*, NCIMB 30273) of the LumiMARA system.

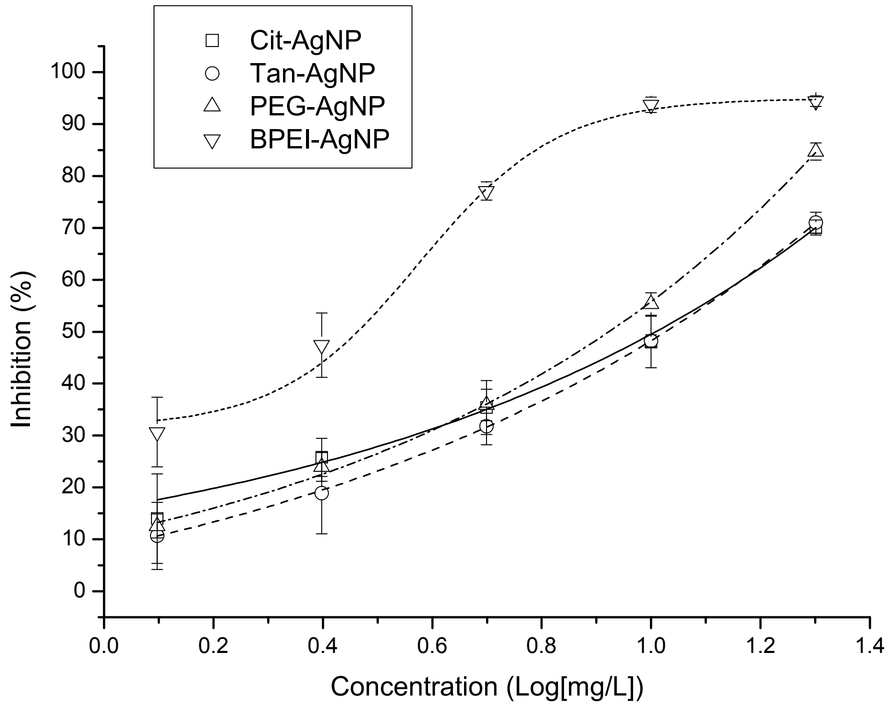

Figure S9. Fitted sigmoidal dose-response curves for the 4 different surface-coated AgNPs with luminescent bacteria strain #9 (*Vibrio fischeri*, NCIMB 30274) of the LumiMARA system.

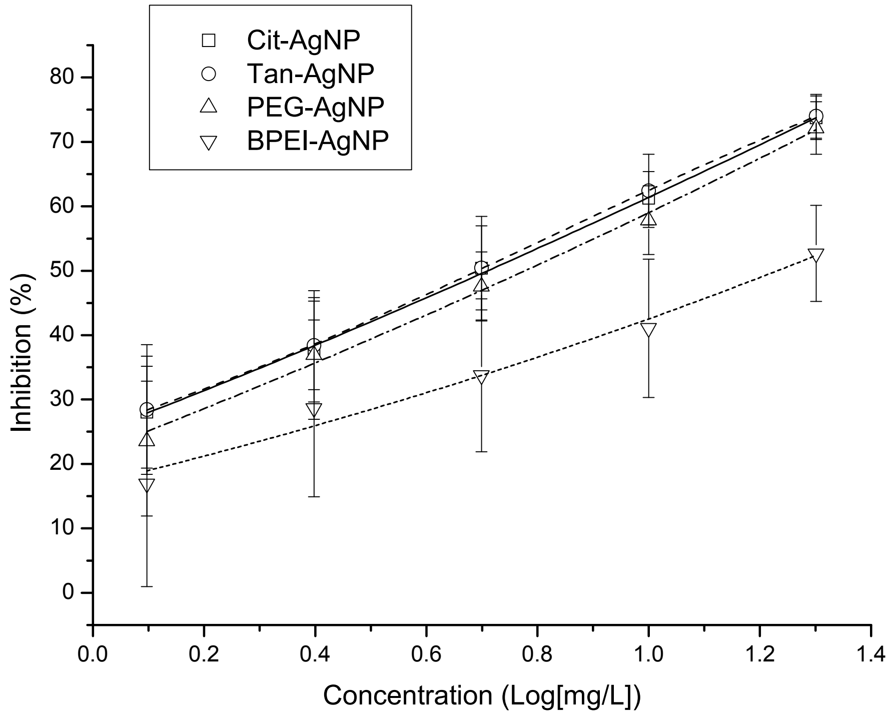

Figure S10. Fitted sigmoidal dose-response curves for the 4 different surface-coated AgNPs with luminescent bacteria strain #10 (*Photorhabdus luminescens*, NCIMB 30275) of the LumiMARA system.

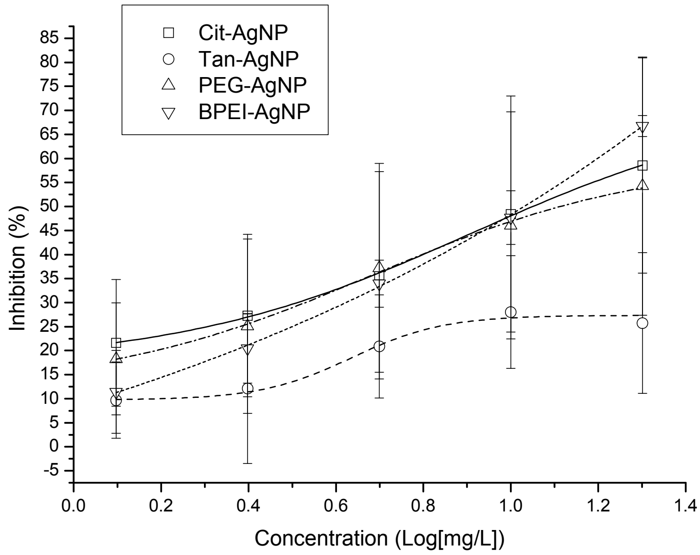

Figure S11. Fitted sigmoidal dose-response curves for the 4 different surface-coated AgNPs with luminescent bacteria strain #11 (*Photorhabdus asymbiotica*, NCIMB 30276) of the LumiMARA system.

S2. Silver Ions Released from Surface Coated AgNPs

The titration method using the ion selective electrode (ISE) is one of the most sensitive and accurate techniques for determining unknown concentrations of compounds in a solution [1]. Silver ion

concentrations released from surface-coated AgNPs were measured using this titration method with silver ISE, purchased from PerfectION, Mettler Toledo, Schwerzenbach, Switzerland. A 0.013 mg/L solution of potassium iodide (1.02×10^{-4} mM as iodide), purchased from Sigma-Aldrich (St. Louis, MO, USA) was used as the titrant and titrated against 0.015 µL volumes of the AgNPs solutions (4.71 mg/L as mass concentration of silver) in an osmotic adjusting solution until reaching the equivalent point. While adding the titrant (volume of 10 µL), the solution was stirred at a uniform rate. The equivalent point was determined at the steepest slope of the titration curve. The concentration of the free silver ions released was calculated by the following equation;

$$C_s = \left({}^{V_t}/_{V_s}\right) C_t$$

where, C_s and C_t are the concentrations of sample and titrant, respectively, and V_s and V_t are the volumes of sample and titrant, respectively.

One of the hypotheses to verify the toxic effect of the AgNPs is the toxicity of the AgNPs results from the free silver ions that are released [1,2]. Among all tested surface-coated AgNPs, however, the determination of the free silver ions released from Cit-AgNPs, Tan-AgNPs, and PEG-AgNPs was not successful by the titration method. It is, therefore, assumed that these three surface-coated AgNPs were stable in the solution and not releasing the free silver ions, and that any effects of the free silver ions from these nanoparticles to the luminescent bacteria was not significant.

Regarding BPEI-AgNPs, the resulting titration curve is shown in Figure S12. The concentration of the free silver ions released from these nanoparticles was calculated as 1.16×10^{-6} mM (1.25×10^{-4} mg/L) from this figure. However, this value is lower than the detection limit of ISE, 0.01 mg/L. Therefore, in this experiment, the effects of free silver ions released from all four surface-coated AgNPs on 11 different luminescent bacteria are limited.

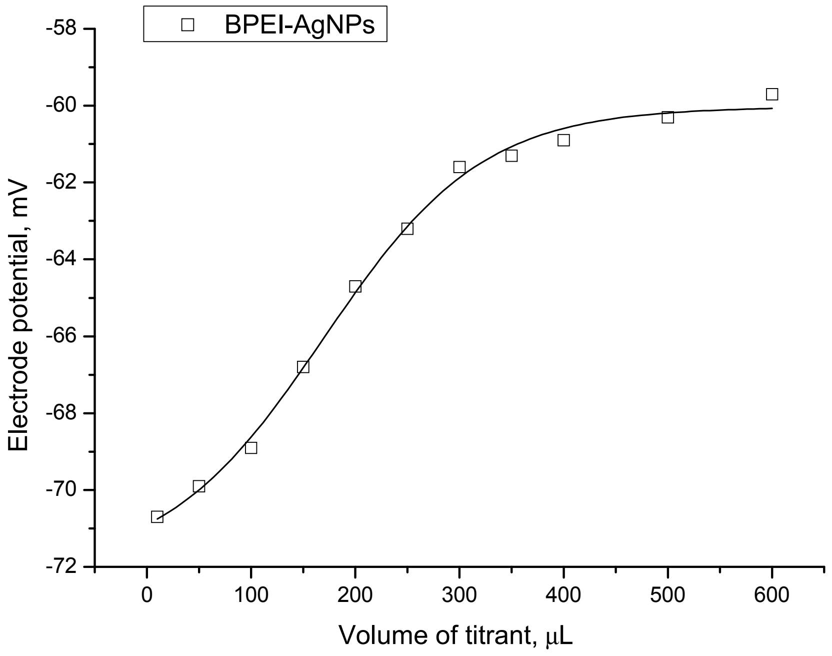

Figure S12. Titration curve of BPEI-AgNPs for determining the free silver ions released from these nanoparticles; titration of iodide (1.02×10^{-4} mM) against 0.015 µL volumes of AgNPs solution (4.71 mg/L as mass concentration of silver).

S3. Stability of Surface-Coated AgNPs in LumiMARA Medium

The exposure time of LumiMARA for tested surface-coated AgNPs was 15 min, and stability test of AgNPs was performed by Malvern Zetasizer Nano ZS at 25 °C with the 173° of a back-scattering angle using 10 mg/L of AgNPs in LumiMARA medium during their exposure time. Cit-AgNPs and Tan-AgNPs were aggregated in 2% NaCl solution, which was used for marine bacteria with Reagent 1, and thus Cit-AgNPs were selected as representative for aggregated nanoparticles in 2% NaCl solution to investigate the stability in the medium of Reagent 2 only. PEG-AgNPs and BPEI-AgNPs, however, were not aggregated in 2% NaCl solution, and BEPI-AgNPs were selected as representative for stable nanoparticles in 2% NaCl solution and tested for the stability in the medium of both Reagent 1 and 2. As a result of this experiment, surface charge of Cit-AgNPs during 15 min of exposure time in Reagent 2 has been slightly changed from −46.5 ± 0.07 mV to −52.8 ± 2.05 mV. In addition, surface charge of BPEI-AgNPs during 15 min of exposure time has also slightly been changed from 11.5 ± 2.52 mV to 12.1 ± 2.14 mV and 31.1 ± 6.35 mV to 35.6 ± 1.63 mV in Reagent 1 and Reagent 2, respectively. Changes in hydrodynamic diameter and polydispersity index (PdI) of those AgNPs were also investigated and the results are shown in Figure S13. From this figure, it is known that all the sizes and PdI of selected AgNPs were very stable during their exposure time in tested medium. Consequently, nanoparticles in tested medium during their exposure time were physically stable enough and the effects of changes in their properties on tested bacteria may be limited.

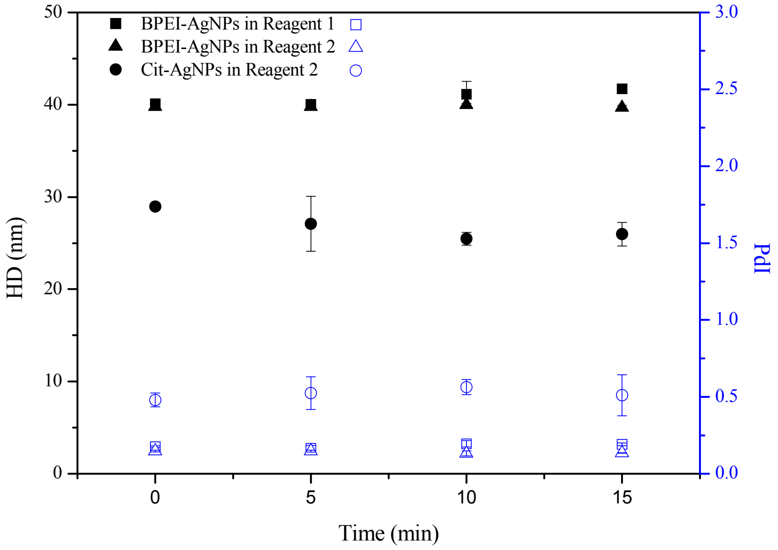

Figure S13. Time-dependent changes in hydrodynamic diameter (HD) and polydispersity index (PdI) for Cit-AgNPs and BPEI-AgNPs during their exposure time in tested medium.

S4. Toxicological Assessment of Surface Coating Materials by LumiMARA

Sodium citrate tribasic dehydrate, tannic acid, poly(ethyleneglycol) methyl ether thiol (average Mn of 6000), and branched polyethylenimine (average Mw of 25,000 and average Mn of 10,000) were purchased from Sigma-Aldrich, St. Lous, MO, USA. The values of the 50% effective concentration (EC_{50}) for bioluminescent bacteria exposed to these four different coating materials were measured following the manufacturer's protocol using Luminous microbial assay for toxicity risk assessment (LumiMARA)

system (NCIMB Ltd., Bucksburn, Aberdeen, UK). The overall method of this experiment was following the same procedure for four surface-coated AgNPs in the main text. Box plots of EC_{50} values of tested luminescent bacteria for four surface coating materials are depicted in Figure S14.

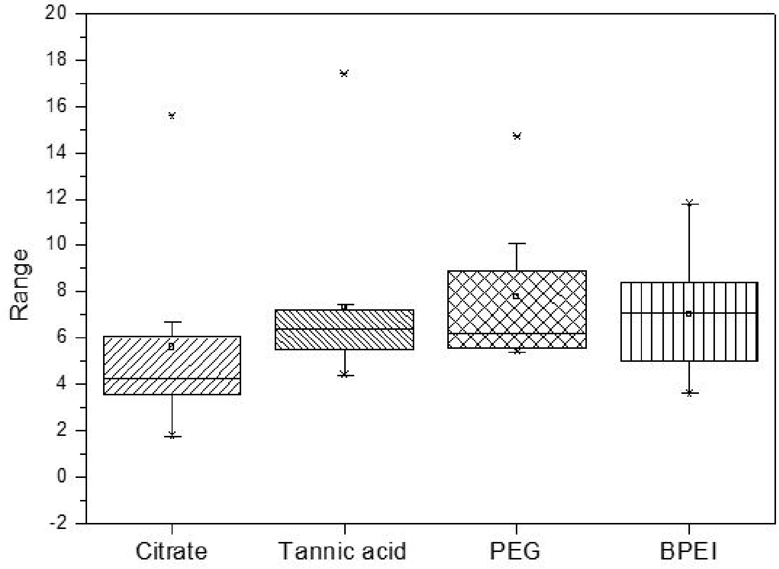

Figure S14. Box plot of average EC_{50} values for four surface coating materials obtained from LumiMARA experiment.

References

1. Koch, M.; Kiefer, S.; Cavelius, C.; Kraegeloh, A. Use of a silver ion selective electrode to assess mechanisms responsible for biological effects of silver nanoparticles. *J. Nanopart. Res.* **2012**, *14*, 1–11.
2. Liu, J.Y.; Sonshine, D.A.; Shervani, S.; Hurt, R.H. Controlled Release of Biologically Active Silver from Nanosilver Surfaces. *ACS Nano* **2010**, *4*, 6903–6913.

© 2015 by the authors; licensee MDPI, Basel, Switzerland. This article is an open access article distributed under the terms and conditions of the Creative Commons Attribution license (http://creativecommons.org/licenses/by/4.0/).
